# Faecal haemoglobin concentration in adenoma, before and after polypectomy, approaches the ideal tumour marker

**DOI:** 10.1177/00045632221080897

**Published:** 2022-03-02

**Authors:** Craig Mowat, Jayne Digby, Shirley Cleary, Lynne Gray, Pooja Datt, David R Goudie, Robert JC Steele, Judith A Strachan, Adam Humphries, Callum G Fraser

**Affiliations:** 1 Department of Gastroenterology, 1251 Ninewells Hospital and Medical School, Dundee, UK; 2 Centre for Research Into Cancer Prevention and Screening, 85326 University of Dundee School of Medicine, Dundee, UK; 3 Department of Surgery, 1251 Ninewells Hospital and Medical School, Dundee, UK; 4 Department of Gastroenterology, St Mark’s Hospital and Academic Institute, London, UK; 5 Department of Genetics, 1251 Ninewells Hospital and Medical School, Dundee, UK; 6 Department of Blood Sciences and Scottish Bowel Screening Laboratory, 1251 Ninewells Hospital and Medical School, Dundee, UK

**Keywords:** Adenoma, colorectal cancer, faecal immunochemical test, faecal haemoglobin, polypectomy, surveillance

## Abstract

**Background:**

Polypectomy may be performed at colonoscopy and then subsequent surveillance undertaken. It is thought that faecal haemoglobin concentration (f-Hb), estimated by quantitative faecal immunochemical tests (FIT), might be a useful tumour marker.

**Methods:**

Consecutive patients enrolled in colonoscopy surveillance were approached at two hospitals. A specimen for FIT was provided before colonoscopy and, ideally after 3 weeks, a second FIT sample from those who had polypectomy. A single FIT system (OC-Sensor io, Eiken Chemical Co., Ltd) was used to generate f-Hb.

**Results:**

1103 Patients were invited; 643 returned a FIT device (uptake: 58.3%). Four patients had known inflammatory bowel disease (IBD) and were excluded, leaving 639 (57.9%) with an age range of 25–90 years (median 64 years), 54.6% male. Of 593 patients who had a f-Hb result and completed colonoscopy, advanced neoplasia was found in 41 (6.9%); four colorectal cancer (CRC): 0.7% and 37 advanced adenoma (AA): 6.3%, and a further 127 (21.4%) had non-advanced adenoma (NAA). The median f-Hb was significantly greater in AA as compared to NAA; 6.0 versus 1.0 μg Hb/g faeces, *p* < 0.0001.134/164 (81.7%) of invited patients returned a second FIT device: 28 were patients with AA in whom median pre-polypectomy f-Hb was 19.2, falling to 3.5 μg Hb/g faeces post-polypectomy, *p* = 0.01, and 106 with NAA had median pre-polypectomy f-Hb 0.8 compared to 1.0 μg Hb/g faeces post-polypectomy, *p* = 0.96.

**Conclusions:**

Quantitative FIT could provide a good tumour marker in post-polypectomy surveillance, reduce colonoscopy requirements and minimise potential risk to patients.

## Introduction

The ideal tumour marker would demonstrate high clinical sensitivity and a low rate of false negative results, high clinical specificity and a low rate of false positive results and would show a positive correlation with both tumour size and stage. The clinical usefulness would have been verified by prospective trials. Analytically, the ideal marker would be quantitative, non-invasive, inexpensive, simple and able to be automated.^1^ Such analyses would be able to be performed under International Organization for Standardization (ISO) 15189 standards to assure quality.^2^

Polypectomy at colonoscopy is viewed as fundamental to effective prevention of colorectal cancer (CRC) and reduces CRC incidence and mortality by altering the natural history and progression of precancerous precursor polyps^3^; studies have shown varying degrees of protection from further neoplasia after polypectomy, highlighting the central importance of adequate surveillance of patients at future risk.^4^ However, the rate of progression of advanced adenoma to CRC is estimated to be low (2.6% in patients aged 50–59 years and 5.6% in those >80 years), and routine colonoscopy surveillance reduces CRC mortality by only 1.7% and increases the number of colonoscopies by 62%.^5^

Much current interest exists in using very low faecal haemoglobin concentrations (f-Hb) in many clinical settings, including CRC screening,^6^ assessment of patients presenting with lower gastrointestinal symptoms^7^ and in adenoma surveillance programmes.^8^ One rationale is that the lower the f-Hb threshold applied to guide further investigation, the more neoplasia will be detected. In addition, if f-Hb could replace colonoscopy, especially in surveillance programmes, scarce endoscopy resources could be saved and patients could benefit from not having to undergo an unpleasant invasive procedure with some, albeit small, risk.^9^

However, compared to CRC screening and detection of significant colorectal disease (SCD = CRC + advanced adenoma [AA] + inflammatory bowel disease [IBD]) in patients with symptoms, the role of f-Hb estimation using faecal immunochemical tests for haemoglobin (FIT) in surveillance following polypectomy remains unclear. It has been stated that further research is warranted to better define potential applications.^8^ In consequence, the aim of this study was to determine, for the first time, using a quantitative FIT at very low f-Hb, as defined by the analytical detectability characteristics,^10^ (a) whether f-Hb had the characteristics of an ideal tumour marker in this clinical setting and (b) whether f-Hb changed following polypectomy in patients at increased risk of CRC and engaged in a surveillance programme.

## Methods

Consecutive patients already enrolled in colonoscopy surveillance were approached at Ninewells Hospital and Medical School, Dundee, Scotland, UK, and St Marks Hospital, London, England, UK. Individuals were contacted at their appointment time by a colorectal nurse specialist and, provided the patient still fulfilled the surveillance criteria described by the British Society of Gastroenterology (BSG) guidelines applicable at the time,^11^ although since updated,^12^ a surveillance colonoscopy was booked. The nurse invited each patient to submit a single sample of faeces for faecal immunochemical test for haemoglobin (FIT) analysis prior to the colonoscopy. A FIT specimen collection device (OC-Sensor, Eiken Chemical Co., Ltd, Tokyo, Japan) and a pictorial patient information sheet were sent to the patient’s home. The sample collection for FIT was completed before bowel preparation started. Samples were returned to Blood Sciences, NHS Tayside, Ninewells Hospital and Medical School, Dundee, Scotland, UK, which is accredited to ISO 15189 standards.^2^ Return of a device was considered as implied consent to take part in the study. The study had ethical approval from NRES North of Scotland ethic committee reference: 14/NS/0059.

Analyses were performed on one OC-Sensor io automated analyser (Eiken Chemical Co., Ltd, Tokyo, Japan). The FIT system provided numerical results for the f-Hb from 0 to >200 μg Hb/g faeces, with a manufacturer’s stated limit of detection (LoD) of 2 μg Hb/g feces and limit of quantitation (LoQ) of 4 μg Hb/g faeces. The manufacturer recommends 10–200 μg Hb/g faeces as the analytical working, or measurement, range. However, because this was a research study and the FIT system used does provide numerical data from 0 μg Hb/g faeces upwards, we used the reported numerical results that were less than the LoD to better investigate the utility of f-Hb in surveillance, as has been done in recent work on f-Hb in screening which clearly demonstrates benefits in the assessment of the future risk of neoplasia in participants with f-Hb below the LoD.^6,13^ Feasibility and acceptability of application of FIT was assessed by uptake. Endoscopists were blind to the f-Hb result and recorded their findings on the appropriate hospital’s electronic endoscopy reporting system. Polyp size and number were verified by a specialist gastrointestinal pathologist. Adenomatous polyps were grouped by size (<10 mm, ≥ 10 mm) and number. Individuals with small rectal hyperplastic polyps were considered as normal. If multiple lesions were present, classification was based on the most advanced lesion. AA was defined as the presence of ≥3 adenoma or any one ≥10 mm.

Those patients who underwent polypectomy were invited to submit a second faecal sample for f-Hb estimation after an interval, ideally, of at least 3 weeks, a time interval arbitrarily chosen, but based on experience as the time required for healing of colorectal lesions due to polypectomy. The f-Hb before and after polypectomy were compared. The time intervals between first and second FIT collections were examined and the relationship between time intervals and observed changes in f-Hb explored. Statistical analyses were performed with MedCalc (MedCalc Software, Ostend, Belgium.

## Results

**Figure 1.**
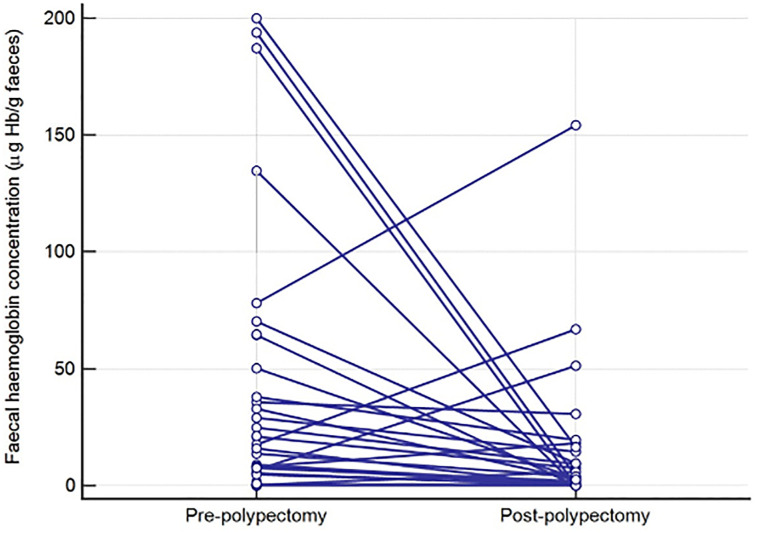
Faecal haemoglobin concentration (μg Hb/g faeces) pre- and post-polypectomy in patients with advanced adenoma.

**Figure 2.**
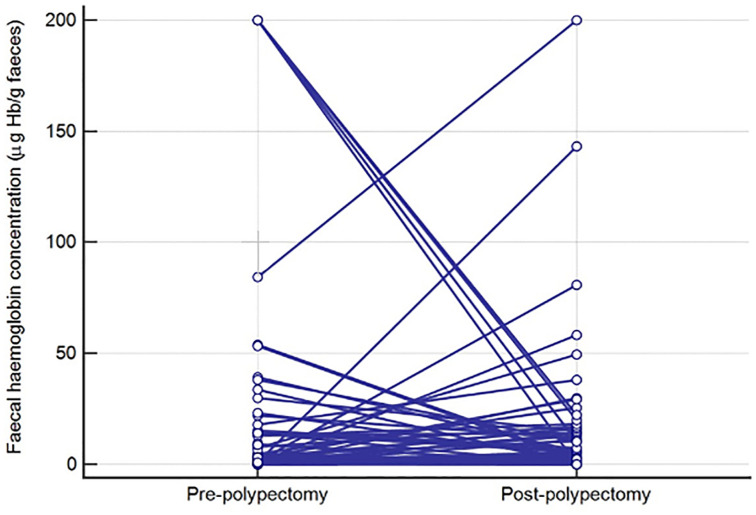
Faecal haemoglobin concentration (μg Hb/g faeces) pre- and post-polypectomy in patients with non-advanced adenoma.

This investigation was conducted within a larger study examining the utility of FIT at the LoD within a cohort at increased risk of CRC who were attending for scheduled surveillance colonoscopy; the study has been described in detail elsewhere.^14^ Briefly, between 01 June 2014 and 30 September 2016 inclusively, 1103 patients were invited, and 643 returned a FIT device (uptake: 58.3%). Four patients had known IBD and were excluded, leaving 639 (57.9%) with an age range of 25–90 years (median 64 years, IQR 55–71), 54.6% male. The indications for colonoscopy reflected routine surveillance practice and were not specifically defined for this study. Of the final 593 patients who had a f-Hb result and completed colonoscopy, advanced neoplasia was found in 41 (6.9%), comprising four CRC (0.7%) and 37 AA (6.3%); a further 127 patients (21.4%) had non-advanced adenoma (NAA). The median f-Hb for all adenomas was 0.8 μg Hb/g faeces (95% CI: 0.4–1.0). The median f-Hb was significantly greater in AA as compared to NAA; 6.0 μg Hb/g faeces (95% CI: 6.7–32.5) versus 1.0 μg Hb/g faeces (95% CI: 0.4–1.8]), *p* < 0.0001). All patients underwent polypectomy at the time of colonoscopy and 134/164 (81.7%) of invited patients returned a second FIT device for f-Hb estimation after 9–69 days, median 28 days (inter-quartile range: 22–35 days). Of the 51 patients who had a higher second f-Hb, 13 had less than 21 days between the tests (25.5.%). Of the remaining 83 who had a lower or identical second f-Hb, 12 had less than 21 days between the two (14.5%). Of the 134 patients with two f-Hb results, 28 were patients with AA in whom median pre-polypectomy f-Hb was 19.2 μg Hb/g faeces (95% CI: 8.1–37.0) falling to 3.5 μg Hb/g faeces (95% CI: 0.5–9.5) post-polypectomy, *p* = 0.01 (Figure 1) and 106 were patients with NAA in whom the median pre-polypectomy f-Hb was 0.8 μg Hb/g faeces (95% CI: 0.4–1.8) compared to 1.0 μg Hb/g faeces (95% CI 0.6–2.0) post-polypectomy, *p* = 0.96 (Figure 2).

## Discussion

We have shown here that, before polypectomy, f-Hb is significantly greater in patients with AA than those with NAA, demonstrating that f-Hb fulfils one of the criteria of a good tumour marker because the f-Hb is related to the size and the stage of lesions. This is in keeping with the findings from many studies of f-Hb in population-based screening, in which f-Hb is higher in AA than NAA and, indeed, f-Hb in NAA is not different to those with normal colonoscopy or other less serious bowel disease.^15,16^ We have also previously documented that f-Hb is related to colorectal disease severity in patients presenting in primary care with lower gastrointestinal symptoms.^17^

In addition, and for the first time to our knowledge, we have demonstrated that, following polypectomy, overall f-Hb fell significantly in patients with AA but not in those with NAA. However, it was found, as shown in Figure 1 and 2, that the f-Hb rose in some patients; 25.5% of these patients had less than 21 days between the two collections. In contrast, of the patients who had a lower or identical second f-Hb result, only 14.5% had less than 21 days between collections. Thus, the post-polypectomy healing might have been incomplete in those who had two collections less than 21 days apart; in consequence, we recommend that the time interval between collections pre- and post-polypectomy should be at least 21 days. However, the finding of raised f-Hb with no unusual findings on colonoscopy is common in screening participants and in patients presenting in primary care,^18^ and the rise in f-Hb seen in some may have been due to a bleed from another less significant colorectal lesion, such as haemorrhoids or inflamed acute diverticulosis/itis, or indeed it is possible that the colonoscopist missed another lesion. However, more importantly, the significant fall in f-Hb following polypectomy in those with AA suggests that the found lesion has been the key arbiter of f-Hb in this clinical setting. A germane question is whether a repeat FIT after a finding of an increase in f-Hb could help decide whether more traditional surveillance protocols with colonoscopy should be initiated, although there is little available evidence on the possible roles of repeat FIT in any clinical setting.^19^ It would also be of interest to investigate if serial f-Hb in patients with NAA and in AA, when f-Hb has fallen, could detect, at an early stage, development of neoplasia; early detection of lesions is another ideal attribute of a tumour marker. Such a strategy is used in IBD to predict recurrence of disease using serial faecal calprotectin measurements (with the patient acting as their own control).^20^

It has been previously suggested that use of FIT in surveillance programmes could assist in assessing the future risk of colorectal neoplasia, and that surveillance of those patients at higher risk of future CRC could use regular FIT, following which, individual patients could be counselled and the surveillance colonoscopy programme directed to those most likely to benefit.^21,22^ Our data suggests that NAA are unlikely to be picked up in adenoma surveillance programmes using FIT but, since these rarely progress to neoplasia, this is not concerning. Perhaps patients under surveillance with undetectable f-Hb in routine practice, that is, f-Hb lower than the LoD, which is lower than the thresholds generally used in CRC screening and assessment of patients with symptoms, could have their surveillance intervals increased, saving colonoscopy resources, which are scarce in many countries. Patients with detectable f-Hb, above the LoD, are more likely to have CRC or AA and therefore, if FIT are used as indicators for colonoscopy in surveillance programmes, we suggest that investigation should not be delayed. It would be of interest, as in screening, to assess if patients with f-Hb less than the LoD, but measurable, have a higher risk that those with f-Hb of 0 μg Hb/g faeces in the subsequent years.

Finally, quantitative FIT, which give estimates of f-Hb, do fulfil many of the requirements of the ideal tumour marker, being non-invasive and with simple to use, hygienic, specimen collection devices.^23^ Further, it has been found that a majority of participants in a hypothetical study stated a preference for a surveillance test resembling FIT over colonoscopy; however, the authors did admit that research should test whether this translates to greater uptake in a real surveillance setting.^24^ And, compared to colonoscopy, FIT are very inexpensive.^4^

## Conclusions

Quantitative f-Hb estimates generated in patients in post-polypectomy surveillance programmes using FIT have many of the merits required of a tumour marker. Their use could reduce colonoscopy requirements in this clinical setting and thereby also reduce the potential risk to patients of this invasive investigation. However, further investigation of a number of aspects is warranted before adoption into routine practice.

## Appendix

### Abbreviations

AA: advanced adenoma
BSG: British Society of Gastroenterology
CI: confidence intervals
CRC: colorectal cancer
FIT: faecal immunochemical tests for haemoglobin
f-Hb: faecal haemoglobin concentration
IBD: inflammatory bowel disease
LoD: limit of detection
LoQ: limit of quantitation
NAA: non-advanced adenoma
NICE: National Institute for Health and Care Excellence
UK: United Kingdom
